# Lactate Accelerates Early Angiogenesis and Bone Regeneration Through Macrophage M1 Polarisation

**DOI:** 10.1111/cpr.70177

**Published:** 2026-01-26

**Authors:** Lulu Liu, Danning Ma, Jia Song, Boon Chin Heng, Ying Huang, Xuehui Zhang, Mingming Xu, Yan Wei, Tai Wei, Jinqi Wei, Xuliang Deng

**Affiliations:** ^1^ First Clinical Division Peking University School and Hospital of Stomatology and National Center of Stomatology and National Clinical Research Center for Oral Diseases and National Engineering Research Center of Oral Biomaterials and Digital Stomatology and Research Center of Engineering and Technology for Computerized Dentistry Ministry of Health and NMPA Key Laboratory for Dental Materials Beijing People's Republic of China; ^2^ Department of Dental Materials Dental Medical Devices Testing Center, Peking University School and Hospital of Stomatology and National Center of Stomatology and National Clinical Research Center for Oral Diseases and National Engineering Research Center of Oral Biomaterials and Digital Stomatology and Research Center of Engineering and Technology for Computerized Dentistry Ministry of Health and NMPA Key Laboratory for Dental Materials Beijing People's Republic of China; ^3^ Department of Geriatric Dentistry Peking University School and Hospital of Stomatology and National Center of Stomatology and National Clinical Research Center for Oral Diseases and National Engineering Research Center of Oral Biomaterials and Digital Stomatology and Research Center of Engineering and Technology for Computerized Dentistry Ministry of Health and NMPA Key Laboratory for Dental Materials Beijing People's Republic of China

**Keywords:** angiogenesis, bone regeneration, lactate, M1 polarisation, macrophages

## Abstract

Failure of timely bone regeneration compromises structural integrity and delays functional recovery; therefore immune regulation of the early repair microenvironment is crucial for successful healing. M1 (pro‐inflammatory) phenotype macrophages play pivotal roles in vascularisation during the early phase of bone regeneration and are typically activated by interferon‐gamma (IFN‐γ) or lipopolysaccharide (LPS) as well as by metabolite‐derived signals. Lactate, a metabolite known to regulate a series of pathophysiological processes, has not yet been fully investigated for its specific immunomodulatory role in the microenvironment of bone injury healing. Our in vitro experiments demonstrated that lactate induced macrophage polarisation to the M1 phenotype and accelerated angiogenesis, with the HIF1α‐NOD1‐calcium influx axis identified as a key mediator. In vivo validation further confirmed the positive effects of lactate intervention in promoting vascularised bone regeneration at the early stage of injury. Thus, this study uncovers how lactate modulates immune response in association with M1 macrophages and indicates its potential as a therapeutic strategy for promoting vascularised bone healing.

## Graphical abstract

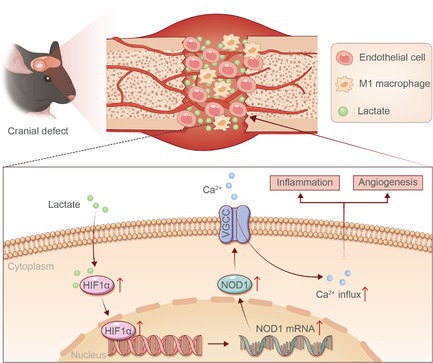

## 1 Introduction

Macrophages play crucial roles in maintaining tissue homeostasis and orchestrating the sequential phases of regeneration [1]. Following bone injury, macrophages are among the earliest immune cells recruited to the defect site, where they undergo rapid polarisation towards the M1 phenotype to initiate inflammatory responses [2]. This early inflammatory phase is indispensable for debris clearance, recruitment of reparative cells and the activation of downstream regenerative cascades [3]. M1 macrophages not only release pro‐inflammatory cytokines such as tumour necrosis factor‐alpha (TNFα), interleukin‐1 beta (IL1β) and interleukin‐6 (IL6), but also secrete high levels of vascular endothelial growth factor (VEGF), which stimulates endothelial cell proliferation and promotes angiogenesis, thereby establishing a vascularised microenvironment favourable for subsequent osteogenesis [4]. Traditionally, M1 polarisation has been attributed to classical stimuli such as IFN‐γ or LPS derived from microbial components. Nevertheless, accumulating evidence indicates that M1‐like macrophages persist even in sterile injury conditions and in *Ifn‐γ*‐deficient models [5, 6], implying that non‐infectious cues within the damaged tissue microenvironment can also drive pro‐inflammatory activation. Therefore, M1 polarisation is not exclusively dependent on pathogen‐associated signals, but may also be regulated by endogenous factors such as metabolites, ions or damage‐associated molecular patterns released during tissue injury.

Most studies on macrophage biology have primarily focused on regulating macrophages polarisation towards the anti‐inflammatory M2 phenotype within specific tissue microenvironments. Inducing M2‐like macrophages has been shown to promote muscle regeneration [7], facilitate bone healing [8, 9], and accelerate diabetic wound repair by enhancing extracellular matrix deposition and tissue remodelling [10]. In contrast, relatively less attention has been given to understanding how metabolic cues contribute to the initiation of the pro‐inflammatory M1 phenotype, despite its indispensable role in the early stages of tissue repair. Emerging evidence suggests that specific metabolites can act as intrinsic signalling molecules to direct macrophage polarisation towards the M1 phenotype. For instance, trimethylamine N‐oxide (TMAO) and succinate, two metabolites enriched in the intestinal microenvironment, have been reported to activate macrophages through pattern recognition receptors and metabolic reprogramming pathways, thereby enhancing the expression of pro‐inflammatory factors [11, 12]. These findings highlight the critical role of metabolic signalling in shaping macrophage phenotypes. However, whether and how metabolites within the bone injury microenvironment regulate M1 polarisation remain largely unexplored. Given the metabolically dynamic nature of bone tissue after injury, identifying such regulatory metabolites could provide novel insights into immune‐mediated mechanisms of bone regeneration.

During the early stages of bone injury, the microenvironment is characterised by rapid tissue hypoxia and a marked increase in glycolysis, resulting in the accumulation of large amounts of lactate together with the infiltration of M1 macrophages [13, 14]. Traditionally considered a metabolic waste product, lactate is now recognised as a bioactive signalling molecule that modulates immune cell behaviour in a context‐dependent manner. In inflammatory tissues, lactate has been shown to activate T cells and enhance the production of pro‐inflammatory cytokines such as IL17 and IFN‐γ, thereby amplifying local immune responses [15, 16]. In contrast, within the tumour microenvironment, lactate accumulation leads to immunosuppression by inhibiting the activation and cytotoxicity of T cells, NK cells and dendritic cells, ultimately facilitating tumour immune evasion [17, 18]. These contrasting findings indicate that lactate serves as a key metabolic signal capable of fine‐tuning immune responses according to the surrounding microenvironment. However, how lactate regulates macrophage polarisation and angiogenic responses during bone repair remains unclear. Given that both lactate accumulation and macrophage infiltration are hallmarks of the early bone injury milieu, elucidating the precise immunomodulatory role of lactate in this context is essential for understanding its potential contribution to bone regeneration.

Here, we found that lactate, which increased significantly during the early phase of bone defect healing, increased nucleotide‐binding oligomerisation domain‐containing 1 (NOD1) expression by stabilising hypoxia‐inducible factor 1‐alpha (HIF1α), which in turn promoted macrophages into the M1 phenotype, thereby facilitating the sprouting of endothelial cells. Furthermore, our in vivo studies confirmed that lactate administration enhanced vascularisation and new bone formation at the bone defect area. Interestingly, these effects only occurred when lactate treatment was applied in the early phase of injury. Hence, our findings enrich the theoretical foundation of metabolites‐driven M1 polarisation of macrophages in the bone injury microenvironment, suggesting that lactate plays crucial roles in modulating macrophage immune responses, angiogenesis and bone regeneration, thus opening a new avenue for the development of novel therapeutic strategies for accelerating the vascularised bone healing process.

## 2 Materials and Methods

### 2.1 Animals and Surgical Procedure

The animal surgical procedure was approved by the Institutional Animal Care and Use Committee of the Peking University (Approval number: LA2023047). All animal experiments were performed in Laboratory Animal Science of Peking University Health Science Center. The C57BL/6N mice (6 weeks old) were purchased from Beijing Vital River Laboratory Animal Technology Co. Ltd. (China). Only male mice were used in this study to ensure consistency across experimental groups [19]. Under anaesthesia, a 3‐mm‐diameter cranial defect was created on one side of the calvaria using the sagittal suture as an anatomical reference, whereas the contralateral side across the sagittal suture was left intact and served as the sham surgery (internal) control. Mice were euthanised at 1 day post‐surgery, and bone tissues were collected.

For implantation experiments, collagen sponges were implanted into the defect according to the following groups: (1) Blank (no implant), (2) NS (sponge with normal saline), (3) Lactate (sponge with sodium lactate) and (4) Nodinitib‐1 (sponge with sodium lactate and Nodinitib‐1). Samples were harvested at 1 day for detecting macrophage polarisation, and at 3, 7 and 14 days for inflammatory and angiogenic factors assessment.

For delayed treatment, lactate or NS sponges were implanted at Day 3 (Group1) or 7 (Group2) post‐injury, and vascularisation was analyzed at Day 14.

For osteogenesis assessment, Bio‐Oss Collagen (Geistlich, Switzerland) with or without sodium lactate was implanted, and bone repair was evaluated at 8 and 12 weeks.

### 2.2 Measurement of Lactate Levels in Bone Tissues

Lactate levels in bone tissue were quantified using a Lactate Assay Kit (MAK065, Sigma, USA). Ground bone samples were lysed, filtered (MRCPRT010, Millipore, USA), and reacted with assay buffer for 30 min. Absorbance at 450 nm was measured to determine lactate concentration according to standard curves.

### 2.3 Real‐Time Quantitative PCR Analysis

Total RNA from bone or cell samples was extracted using TRIzol Reagent (Invitrogen, USA), reverse‐transcribed (Takara, Japan), and analyzed with SYBR Green Master Mix (Roche, Germany) on a QuantStudio 3 system (Applied Biosystems, USA). Primer sequences are listed in Table S1.

### 2.4 Enzyme‐Linked Immunosorbent Assay (ELISA)

The bone samples were fully grounded into powder form, then followed by the addition of Tissue Lysis Buffer (abs9225, absin, China). The supernatant was harvested, and the ELISA assays were carried out using the TNFα ELISA kit (abs520010, absin, China) and the IL10 ELISA kit (abs520005, absin, China). The absorbance values were measured at 450 nm, and the concentrations of the TNFα and IL10 proteins were calculated based on a standard curve.

For cell samples, the supernatant of the culture medium or lysate was collected, and subsequent experimental steps were performed as described above. The following kits were used: the NOD1 ELISA kit (MBS9330264, MyBioSource, USA), the TNFα ELISA kit (abs520010, absin, China), and the IL1β ELISA kit (abs520001, absin, China).

### 2.5 Histological Staining

For immunohistochemical staining: decalcified paraffin sections (5 μm) were deparaffinised, subjected to antigen retrieval, blocked, incubated with primary and HRP‐secondary antibodies, visualised using DAB, counterstained with haematoxylin, and sealed for microscopic analysis.

For haematoxylin and eosin (H&E) staining: After sections were deparaffinised and rehydrated, the nuclei were stained using haematoxylin and the cytoplasm was stained with eosin, followed by dehydration and sealing.

For multiplex immunohistochemical (mIHC) staining: All the staining steps were the same as the IHC steps until the addition of the secondary antibody; the mIHC staining kit (NEFP4100, HISTOVA, China) was used according to the manufacturer's instructions.

The following antibodies were used: anti‐TNFα (ab1793, abcam, UK), anti‐VEGF (ab1316, abcam, UK), anti‐CD86 (ab234401, abcam, UK), anti‐CD31 (ab182981, abcam, UK), Donkey Anti‐Rabbit IgG H&L (ab150076, abcam, UK), Goat Anti‐Rabbit IgG H&L (ab6721, abcam, UK) and Rabbit Anti‐Mouse IgG H&L (ab6728, abcam, UK).

### 2.6 Cell Culture

For lactate stimulation, primary mouse bone marrow‐derived macrophages (BMDMs) (CP‐M141, Procell, China) were cultured in high‐glucose Dulbecco's Modified Eagle Medium (DMEM) containing 0, 5 or 30 mM lactate for 24 h.

For the indirect co‐culture of BMDMs with mouse aortic endothelial cells (AECs), the supernatants of BMDMs in the con, 5 mM and 30 mM groups were collected after 24 h of culture, mixed with equal volumes of basal medium (CM‐M075, Procell, China) to generate conditioned medium (CM), and then added to primary AECs (CP‐M075, Procell, China) for functional experiments.

For inhibitor studies, BMDMs were pretreated with 10 μM Nodinitib‐1 (HY‐18639, MedChemExpress, USA) or 20 μM nifedipine (HY‐B0284, MedChemExpress, USA) for 2 h before lactate stimulation. The supernatant was collected and mixed with the basal medium in equal volumes and added to AECs for subsequent experiments.

### 2.7 Flow Cytometry Analysis

Cells were fixed with 4% paraformaldehyde, blocked with 5% BSA, and stained with fluorescent antibodies against CD86 (553691, BD Pharmingen, USA), CD206 (565250, BD Pharmingen, USA), or CD34 (560233, BD Pharmingen, USA). Data were collected on a FACS Calibur system and analyzed using the FlowJo software.

### 2.8 Immunofluorescence

Cells cultured on coverslips were fixed, permeabilised, blocked, and incubated with primary antibodies and fluorescent secondary antibodies. Cytoskeleton and nuclei were stained with Phalloidin (Solarbio, China) and DAPI (Sigma‐Aldrich, USA), respectively. Images were captured using a confocal microscope and analysis was performed using the LAS X software. The following antibodies were utilised: anti‐TNFα (ab1793, abcam, UK), anti‐iNOS (ab178945, abcam, UK), anti‐CD34 (ab81289, abcam, UK), Goat Anti‐Rabbit IgG H&L (ab150077, abcam, UK), and Donkey Anti‐Rabbit IgG H&L (ab150076, abcam, UK).

### 2.9 Tube Formation Assay

The 24‐well plate was placed on ice and coated with 250 μL of pre‐cooled Matrigel without introducing air bubbles and incubated at 37°C for 30 min to allow Matrigel (354234, Corning, USA) to solidify. Next, AECs were digested using trypsin, counted, and resuspended in culture medium, adjusted to a cell density of 1 × 10^5^, and then seeded onto the Matrigel‐coated plate. 500 μL of the DMDM high‐glucose medium or CM was added, according to the aim of the experiment. The 24‐well plate was incubated at 37°C within a 5% CO_2_ incubator, and after 4 h, the tube structures were photographed under a phase contrast microscope. The number of junctions, total tubule length and number of tubules were analyzed using the Image‐Pro Plus software.

### 2.10 Spheroid‐Based Sprouting Angiogenesis Model

2% (w/v) agarose was heated until melted and added to a 3D Petri dish (Microtissues Inc., USA). After solidification, the agarose was peeled out of the 3D Petri dish and placed into six‐well plates for AECs culture. Then 200 μL of AECs suspension was seeded onto the agarose moulds, and 30 min later, the culture medium was added and cellular aggregates were allowed to form. After 12 h, AECs formed spheroids and then the spheroids were embedded in gelatin methacryloyl (GelMA) gels (EFL, China) and light‐cured. The culture medium was added to enable sprouting to continue for 12 h, then the sprouting was photographed using a phase contrast microscope and quantified using the Image‐Pro Plus software to calculate the length of each spheroid, which was recorded as the migration distance.

### 2.11 Measurement of Nitric Oxide (NO) Levels

BMDMs treated with lactate were lysed using Cell Lysis Buffer (S3090, Beyotime Biotechnology, China), and NO levels were measured by the Griess method [20] using a Total NO Assay Kit (S0023, Beyotime Biotechnology, China). Absorbance was read at 540 nm to evaluate the concentration of NO.

### 2.12 RNA Sequencing

BMDMs treated with 0 or 30 mM lactate for 24 h were processed for RNA‐seq. Total RNA was extracted, poly(A) mRNA enriched, fragmented, reverse‐transcribed and sequenced on the Illumina PE150 platform. Differential gene expression was analysed bioinformatically.

### 2.13 Western Blot Assay

The proteins were extracted using RIPA lysis buffer (89901, Thermo Fisher Scientific, USA) and protease inhibitor cocktail (Thermo Fisher Scientific, USA). The protein concentration was measured using a BCA Protein Assay Kit (23227, Thermo Fisher Scientific, USA), and SDS sample loading buffer (P0015F, Beyotime, China) was added to the protein samples, which were then boiled for 10 min at 100°C to denature the proteins. 30 μg of protein samples were then separated by electrophoresis on a 10% (w/v) sodium dodecyl sulphate polyacrylamide gel before being transferred to a polyvinylidene difluoride (PVDF) membrane. Next, the PVDF membrane was blocked with 5% (w/v) skimmed milk for 2 h. Subsequently, the PVDF membrane was cut into different bands according to molecular weight, and then incubated with primary antibody at 4°C overnight. The following day, the bands were incubated with horseradish peroxidase (HRP)‐coupled secondary antibody for 2 h. Finally, the protein bands were visualised using the ECL Western Blot Kit (CoWin Bio., China). The following antibodies were used: anti‐HIF1α (ab179483, abcam, UK), anti‐HIF1β (ab270520, abcam, UK), anti‐CaV1.2 (abs147035, absin, China), anti‐GAPDH (#3683, Cell Signalling Technology, USA) and HRP‐labelled IgG (A0208, Beyotime, China).

### 2.14 Dual Luciferase Reporter Assay

The dual luciferase reporter assay was performed as described previously [21]. Briefly, 293 T cells were co‐transfected with various psiCHECK2 luciferase reporter plasmids and then lysed. Luciferase activity was evaluated using the Dual‐Luciferase Reporter Assay system (Promega, USA), and firefly luciferase activity was normalised to *Renilla* luciferase activity.

### 2.15 siRNA Transfection

According to the manufacturer's instructions, BMDMs were transfected with *NOD1* or scramble siRNA (Ribobio, China). After 24 h, gene silencing efficiency was verified by RT‐qPCR. siRNA sequences are shown in Table S2.

### 2.16 Calcium Mobilisation

For calcium ion imaging experiments, the Fluo‐4 Calcium probe (F14217, Thermo Fisher Scientific, USA) was diluted at a ratio of 1:200 and incubated with BMDMs for 30 min away from light. Subsequently, the BMDMs were continuously photographed using a laser scanning confocal microscope for 4 min. Next, 30 mM lactate was added and continued to be photographed for another 4 min to record changes in the fluorescence intensity of the Fluo‐4 probe.

For the flow cytometry assay, the BMDMs were incubated with the Fluo‐4 Calcium probe (at a dilution ratio of 1:2000) for 20 min away from light. Then, the BMDMs were treated with different reagents. The calcium ion concentration of the cells was measured by flow cytometry, and the mean fluorescence intensity was analysed using the FlowJo software.

### 2.17 Measurement of PKA Activity

PKA activity was determined using the ELISA‐based PKA Kinase Activity Kit (ADI‐EKS‐390A, Enzo Life Sciences, USA) following the manufacturer's instructions. Absorbance at 450 nm was measured, and enzyme activity calculated from standard curves.

### 2.18 OCTA Analysis

The mice were anaesthetised and then fixed in the stereotaxic apparatus to expose the skull. Next, the cranial neovascular network in each group was visualised using a Monitoring System of Vascular Microcirculation in vivo (Micro‐VCC, OPTOPROBE, UK). The reconstructed vascular images were obtained by using the ReconstructionUI software, with different colours of blood vessels representing the different depth locations: red for the deep layer blood vessels, green for the shallow layer, and yellow for the middle layer. The vascular signals in the target area were analyzed using the VesQuant software.

### 2.19 New Bone Regeneration Analysis

At 8 and 12 weeks, skull samples were fixed with 4% (w/v) paraformaldehyde and scanned using a micro‐CT scanner (Inveon, Siemens, Germany). Reconstructed images were analyzed for bone volume per tissue volume (BV/TV) and bone mineral density (BMD) using Inveon Research Workplace software.

### 2.20 Statistical Analysis

Data are presented as mean ± SEM. Comparisons between two groups used unpaired Student's *t*‐test; multiple groups used one‐way ANOVA with Tukey's post hoc test. Statistical significance was set at *p* < 0.05. Graphs were generated using GraphPad Prism.

## 3 Results

### 3.1 Increased Lactate Production Is Spatiotemporally Synchronised With Inflammation and Vascularisation at the Onset of Bone Injury

**FIGURE 1:**
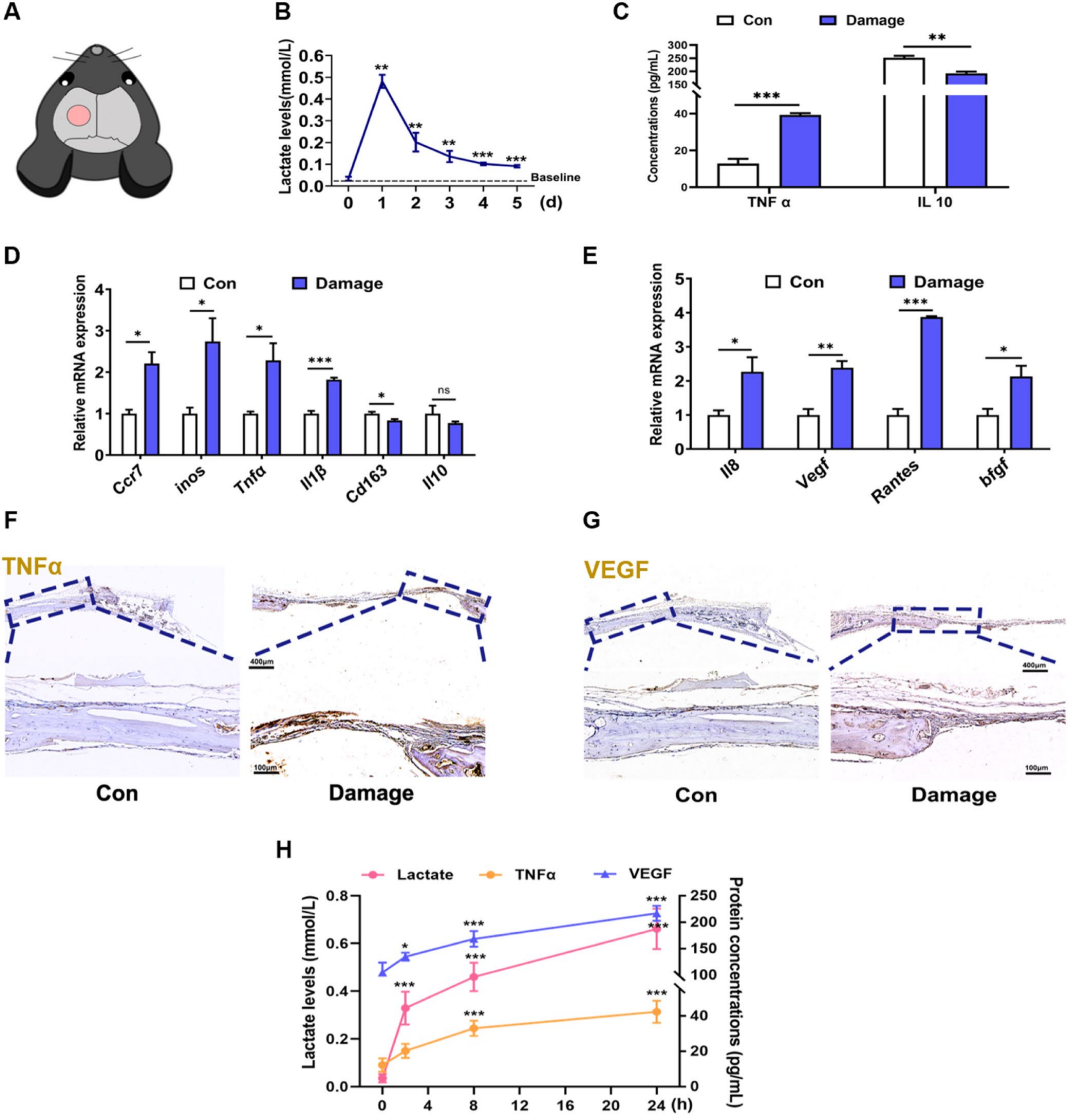
The increase in lactate concentration is spatiotemporally synchronised with inflammation and angiogenesis at the onset of bone injury. (A) Schematic illustration of cranial defect model in mice. (B) The changes in lactate concentrations from the onset of bone injury to day 5 (*n* = 3). (C) The protein concentration of TNFα and IL10 in the cranial bone defect (damage) group and control (con) group at 24 h (*n* = 3). (D) The mRNA expression levels of pro‐inflammatory and anti‐inflammatory genes in the damage and con group at 24 h after surgery (*n* = 3). (E) The mRNA expression levels of angiogenesis‐related genes in the damage and con group at 24 h (*n* = 3). (F, G) IHC staining for detection of TNFα (F) and VEGF (G) in cranial bone defect and intact bone (*n* = 6). Scale bar: Up 400 μm, down 100 μm. (H) The concentration changes of lactate, TNFα, and IL10 at 24 h after bone injury (*n* = 3). All statistical data are presented as mean ± SEM. Samples were subjected to two‐tailed unpaired Student's *t*‐test. Ns, no significance (*p* > 0.05), *p < 0.05, **p < 0.01, ***p < 0.001.

To explore the key features of lactate production from the onset of bone trauma, we constructed a mouse cranial defect model (Figures 1A and S1A) and the cranial tissue was analyzed. The results showed that lactate production increased immediately after surgery and underwent a rapid increase from 0 to 24 h within the defect area that reached a peak, which was 16 times higher than the baseline, followed by a slow decline to about 3 times higher than the baseline at 5 days after injury (Figure 1B). The inflammatory response of M1 macrophages plays a crucial role during the early phase of bone healing [2]. We then tested the inflammatory signals in cranial defects, and the ELISA experiment confirmed that the secretion of TNFα protein increased, as the secretion of interleukin‐10 (IL10) protein decreased at 24 h post‐injury (Figure 1C). Moreover, the M1 pro‐inflammatory markers including *Tnfα, C‐C chemokine receptor 7 (Ccr7), inducible nitric oxide synthase (inos)* and *Il1β* were upregulated, whereas the M2 anti‐inflammatory markers including *Il10*, and *cluster of differentiation 163 (Cd163)* were suppressed in bone defects at 24 h post‐injury, as detected by real‐time quantitative PCR (RT‐qPCR) (Figure 1D).

In order to detect the local vascularisation status of the bone damage area, we analyzed samples from the cranial bone defect at 24 h after surgery and found that the expression of angiogenic‐related cytokines including *Vegf, interleukin‐8 (Il8), regulated on activation, normal T‐cell expressed and secreted (Rantes)*, and *basic fibroblast growth factor* (*bfgf*) was upregulated, as compared with the control group (Figure 1E). Additionally, the immunohistochemistry (IHC) assay also revealed elevated expression levels of TNFα and VEGF within the vicinity of the bone defect region (Figures 1F,G and S1B). We further examined changes in lactate levels, TNFα concentrations, and VEGF concentrations within 24 h of the onset of bone injury, with the line chart showing that increased local lactate levels were accompanied by enhanced concentrations of TNFα and VEGF, suggesting a spatiotemporal correlation between elevated lactate concentration, inflammatory response, and initiation of angiogenesis during the early phase of bone healing (Figure 1H).

### 3.2 Lactate Modulates M1 Polarisation of Macrophages and Also Facilitates Angiogenesis

**FIGURE 2:**
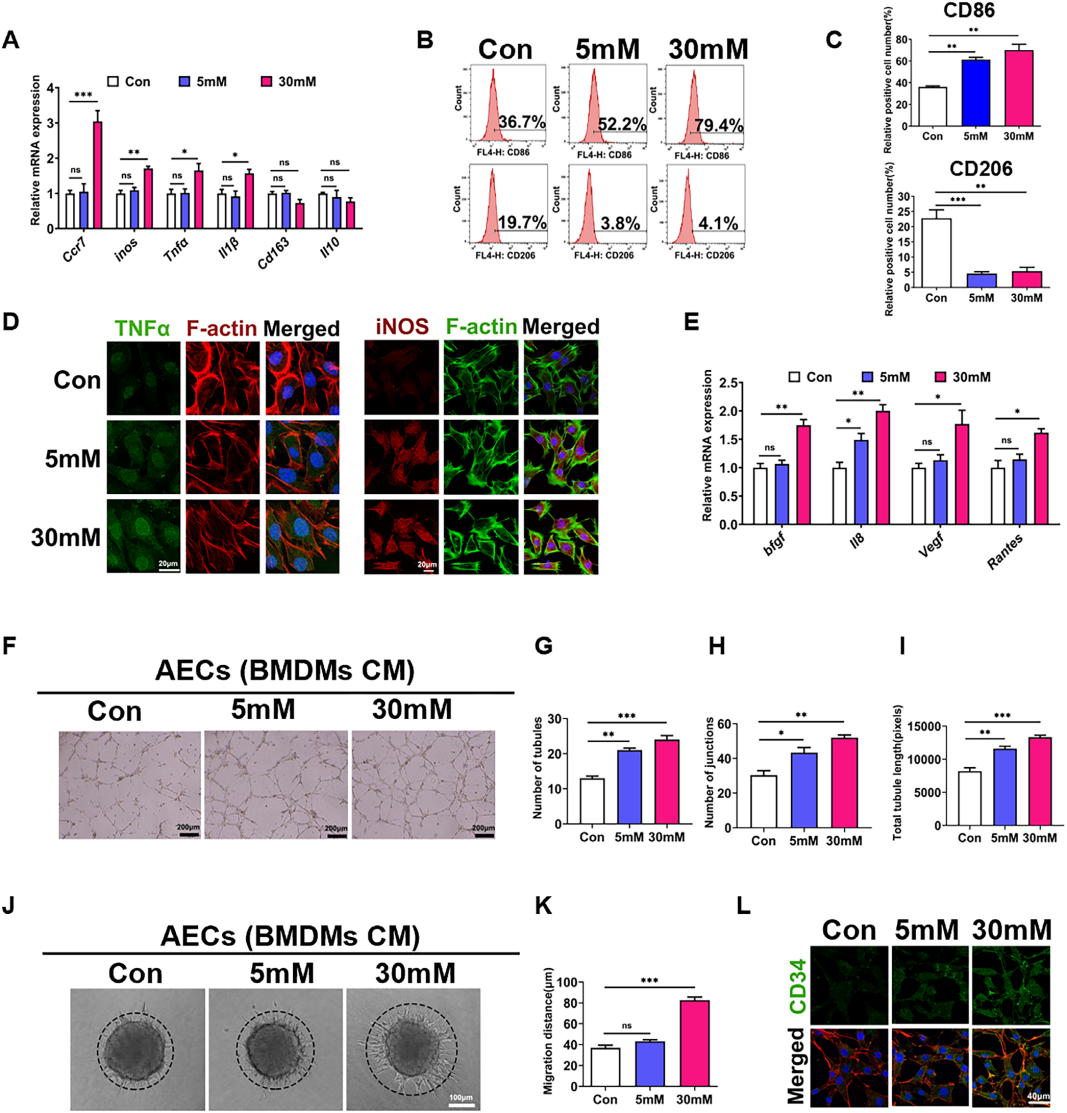
Lactate induces M1 polarisation of macrophages and promotes angiogenesis. (A) RT‐qPCR analysis of the mRNA expression levels of pro‐ and anti‐inflammatory genes after lactate treatment of BMDMs for 24 h (*n* = 3). (B) Flow cytometry analysis of M1 marker CD86 expression levels and M2 marker CD206 expression levels in BMDMs after lactate treatment for 24 h. (C) Quantification statistics of the relative positive cell number from flow cytometry (*n* = 3). (D) Immunofluorescence staining for detection of TNFα and iNOS in BMDMs after 24 h of lactate stimulation. Left panels: TNFα (green), F‐actin (red), and nuclear DNA (blue). Right panels: iNOS (red), F‐actin (green), and nuclear DNA (blue). Scale bar: 20 μm. (E) RT‐qPCR analysis of mRNA expression levels of angiogenesis‐related markers after lactate treatment of BMDMs for 24 h (*n* = 3). (F) Representative images of tube formation at 4 h after AECs were seeded on Matrigel. The supernatant of BMDMs treated with lactate for 24 h was used as the CM to culture AECs. Scale bar: 200 μm. (G–I) The number of tubules, the number of junctions and total tubule length in each group in (F) were quantified using Image Pro Plus software (*n* = 3). (J) Representative photomicrographs of spheroid sprouting assay after AECs were seeded. Scale bar: 100 μm. (K) Quantification statistics of the AECs migration distance (*n* = 3). (L) Immunofluorescence images of CD34 after AECs were treated with BMDMs CM. Cytoskeletal actin, red; nuclear DNA, blue. Scale bar: 40 μm. Results are presented as means ± SEM. Samples were subjected to one‐way ANOVA with Tukey's post hoc test. Ns (*p* > 0.05), *p < 0.05, **p < 0.01, ***p < 0.001.

To further validate the effects of lactate on the pro‐inflammatory functions of macrophages, we treated bone marrow‐derived macrophages (BMDMs) with 5 and 30 mM of sodium lactate for 24 h. The qPCR analysis showed that the expression of M1 phenotype markers *Ccr7, inos, TNFα* and *Il1β* mRNA levels were all upregulated after lactate treatment in the 30 mM group (Figure 2A). Moreover, lactate‐stimulating BMDMs demonstrated a lower percentage of CD206^+^ (M2‐specific marker) cells and a higher percentage of CD86^+^ (M1‐specific marker) cells than the control group (Figure 2B,C). The immunofluorescence results demonstrated that lactate enhanced the staining intensity of TNFα and iNOS in a concentration‐dependent manner (Figure 2D). These findings thus confirmed that lactate enhanced polarisation of BMDMs towards the pro‐inflammatory M1 phenotype.

The expression levels of genes involved in the initiation of angiogenesis by lactate‐treated BMDMs were analyzed. The qPCR assay confirmed the elevated expression levels of *bfgf, Il8, Vegf* and *Rantes* after treatment with 30 mM lactate (Figure 2E). Moreover, NO, which is not only a product of M1 phenotype macrophages but which has also been reported to stimulate angiogenesis in previous studies [20], were also elevated after lactate treatment (Figure S2). M1 phenotype macrophages mediate angiogenesis during the early stages of the healing process via paracrine pathways involving the secretion of angiogenic factors [22]. Then the paracrine effects of lactate‐treated BMDMs on AECs were investigated. The CM for AECs culture was the supernatant of BMDMs treated with or without lactate for 24 h. As a result, the capacity of AECs to form lumens was improved by the administration of CM‐containing lactate, with the 30 mM group yielding the most lumens formation (Figure 2F–I). Once angiogenesis is initiated, specialised endothelial cells denoted as tip cells facilitate capillary sprouting. It is generally regarded that the crucial point in angiogenesis is the specialisation of tip cells [23]. We next explored whether lactate‐treated BMDMs could positively modulate sprouting angiogenesis by modulating the specialisation of endothelial tip cells. The spheroid‐based sprouting angiogenesis model was treated with the CM described earlier, and as shown in Figure 2J, the migration distance of the tip cells from AEC spheroids stimulated with 30 mM CM was much longer than that stimulated with 5 mM CM and con CM (Figure 2J,K). Consistently, the immunofluorescence results showed that the fluorescence intensity of CD34, a marker of tip cells [24], was significantly higher in the 30 mM CM group, as compared to the other groups (Figure 2L). Taken together, these data suggested that lactate promoted in vitro angiogenesis by mediating M1 polarisation in BMDMs at 24 h, which was consistent with the results of the above in vivo experiments.

### 3.3 Lactate Promotes NOD1 Expression by Stabilising HIF1α to Modulate M1 Polarisation in BMDMs

**FIGURE 3:**
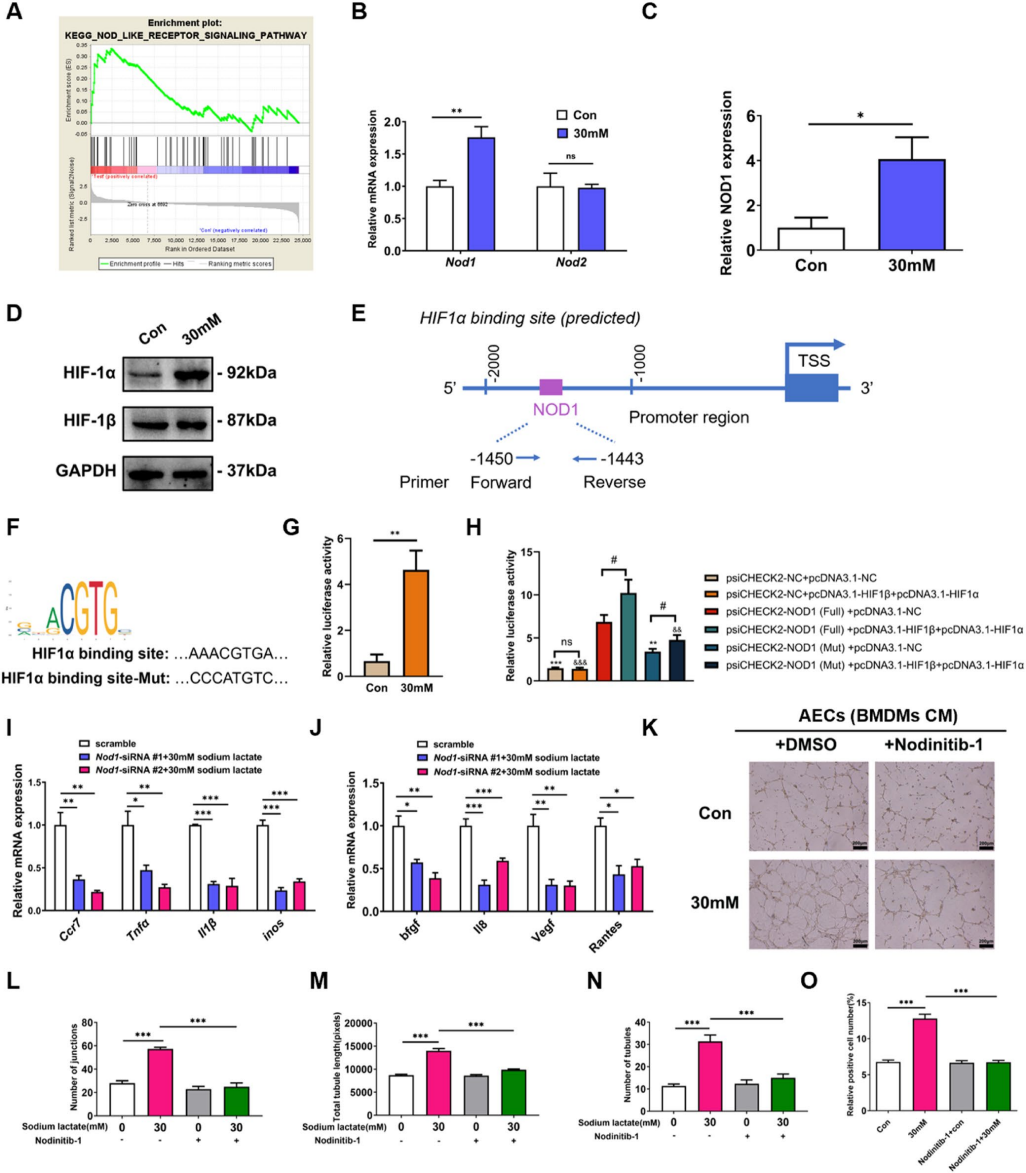
Lactate promotes NOD1 expression by stabilising HIF1α to modulate M1 polarisation of BMDMs.(A) GSEA of differentially expressed genes in BMDMs stimulated with or without 30 mM lactate for 24 h. (B) RT‐qPCR analysis of the mRNA expression levels of *NOD1* and *NOD2* in BMDMs after 24 h treatment with or without lactate (*n* = 3). (C) ELISA assay of the relative NOD1 protein expression in BMDMs after treatment with 30 mM lactate (*n* = 3). (D) Western blot analysis of HIF1α and HIF1β expression in BMDMs treated with or without 30 mM lactate for 24 h. (E) JASPAR predicts a putative HIF1α binding site located on the promoter region of mouse *NOD1*. (F) Sequence analysis of the NOD1 promoter and corresponding mutations. (G) NOD1 promoter‐driven luciferase activity was induced with lactate treatment (*n* = 3). (H) NOD1 promoter (Full and Mut) luciferase activity was detected by dual‐luciferase assays. Ns (*p* > 0.05), **p < 0.01 and ***p < 0.001 compared with psiCHECK2‐NOD1 (Full) + pcDNA3.1‐NC group, ^&&^ *p* < 0.01 and ^&&&^ *p* < 0.001 compared with psiCHECK2‐NOD1 (Full) + pcDNA3.1‐HIF1β + pcDNA3.1‐HIF1α group, ^#^ *p* < 0.05 compared with psiCHECK2‐NOD1 (Full) + pcDNA3.1‐NC group or psiCHECK2‐NOD1 (Mut) + pcDNA3.1‐NC group (*n* = 3). (I, J) Knockdown of *NOD1* by siRNA attenuated the inflammation (I) and pro‐angiogenic effects of lactate (J) (*n* = 3). (K) Representative images of tube formation at 4 h after AECs were seeded on Matrigel. The BMDMs were pre‐treated with or without Nodinitib‐1 for 2 h, then lactate was added, and the BMDMs supernatant was collected as the CM after 24 h. Scale bar: 200 μm. (L–N) The number of junctions, total tube length, and the number of tubules in each group in (K) were quantified using the Image Pro Plus software (*n* = 3). (O) Quantitative flow cytometry statistics of CD34^+^ levels in AECs after treatment with BMDMs CM with or without Nodinitib‐1 (*n* = 3). All statistical data are presented as mean ± SEM. Samples were subjected to one‐way ANOVA and two‐tailed unpaired Student's *t*‐test. Ns (*p* > 0.05), *p < 0.05, **p < 0.01.

RNA sequencing (RNA‐seq) was carried out on BMDMs with or without 30 mM lactate treatment to gain deeper insights into the underlying mechanisms. Differentially expressed genes analysis between the 30 mM group and the con group revealed significant differences in gene expression, indicating that lactate profoundly impacted the physiological functions of BMDMs (Figure S3A). The volcano plot highlighted the upregulation of *Il15, Il18* and *Il1r1*, key regulators of the pro‐inflammatory response [25], in the 30 mM group. Besides, *Cd34* expression was also elevated (Figure S3B). Gene ontology (GO) analysis showed that the enriched biological process (BP) terms of the 30 mM group were mainly in three classes: the inflammatory response, angiogenesis, and the rise in calcium concentration (Figure S3C). Then, we performed gene set enrichment analysis (GSEA) to further validate the contribution of lactate to the modulation of inflammatory and angiogenic responses, and genes involved in NOD‐like receptor (NLR) signalling were higher enriched in 30 mM lactate‐treated cells compared to control cells (Figure 3A).

Given that, we evaluated the expression of NOD1 and NOD2 in BMDMs cultured with 30 mM lactate. The qPCR results indicated that *NOD1* displayed significant upregulation after lactate treatment compared to *NOD2* (Figure 3B). Meanwhile, *NOD1* expression was also elevated in the damaged bone tissues (Figure S3D). Similarly, ELISA analysis showed that 30 mM lactate treatment upregulated NOD1 protein levels (Figure 3C). These findings thus demonstrated that NOD1 was activated by lactate in BMDMs.

The ubiquitination and degradation of HIF1α is attenuated by the lactate‐rich microenvironment, which can mediate angiogenesis by maintaining HIF1α stability. HIF1α enters the nucleus to form dimers with HIF1β to promote gene transcription [26, 27]. Accordingly, the western blot results confirmed the increased protein expression levels of HIF1α in BMDMs after treatment with lactate (Figure 3D). Utilising a transcription factor binding site analysis tool, we identified a potential HIF1α binding site on the NOD1 promoter region (Figure 3E,F). Then we performed the dual‐luciferase assays and examined the activity of the NOD1 promoter region. The results showed that there was enhanced luciferase activity of NOD1 promoter after 30 mM lactate treatment (Figure 3G). The dual‐luciferase assays also confirmed that HIF1α bound to the mouse NOD1 promoter region and induced the transcription of NOD1. The mutation of the HIF1α binding site conversely inhibited the promotion effect of HIF1α on NOD1 transcription (Figure 3H). The above results thus demonstrated that lactate acted as a positive regulator of NOD1 expression by stabilising the transcription factor HIF1α.

Next, siRNA was used to knock down *NOD1* in BMDMs to validate its modulatory effects on downstream biological processes. As shown in Figure 3I,J, the enhanced expression of lactate‐induced inflammation and angiogenesis factors was mitigated after *NOD1* knock down (Figures S3E and 3I,J). Likewise, BMDMs were pre‐treated with the NOD1 inhibitor Nodinitib‐1 for 2 h before 30 mM lactate was administered for 24 h. Supernatant was collected as CM to treat AECs and the tube formation assay confirmed that when NOD1 expression was inhibited, subsequent administration of lactate could no longer improve tube formation, including a reduction in the number of junctions, total tube length, and the number of tubules (Figure 3K–N). The number of CD34^+^ AECs was also decreased by flow cytometry analysis (Figures S3F and 3O). Hence, the results suggest that lactate promoted NOD1 expression by stabilising HIF1α, inducing the M1 polarisation of BMDMs and angiogenesis.

### 3.4 Lactate Activates Calcium Influx in a NOD1‐Dependent Manner

**FIGURE 4:**
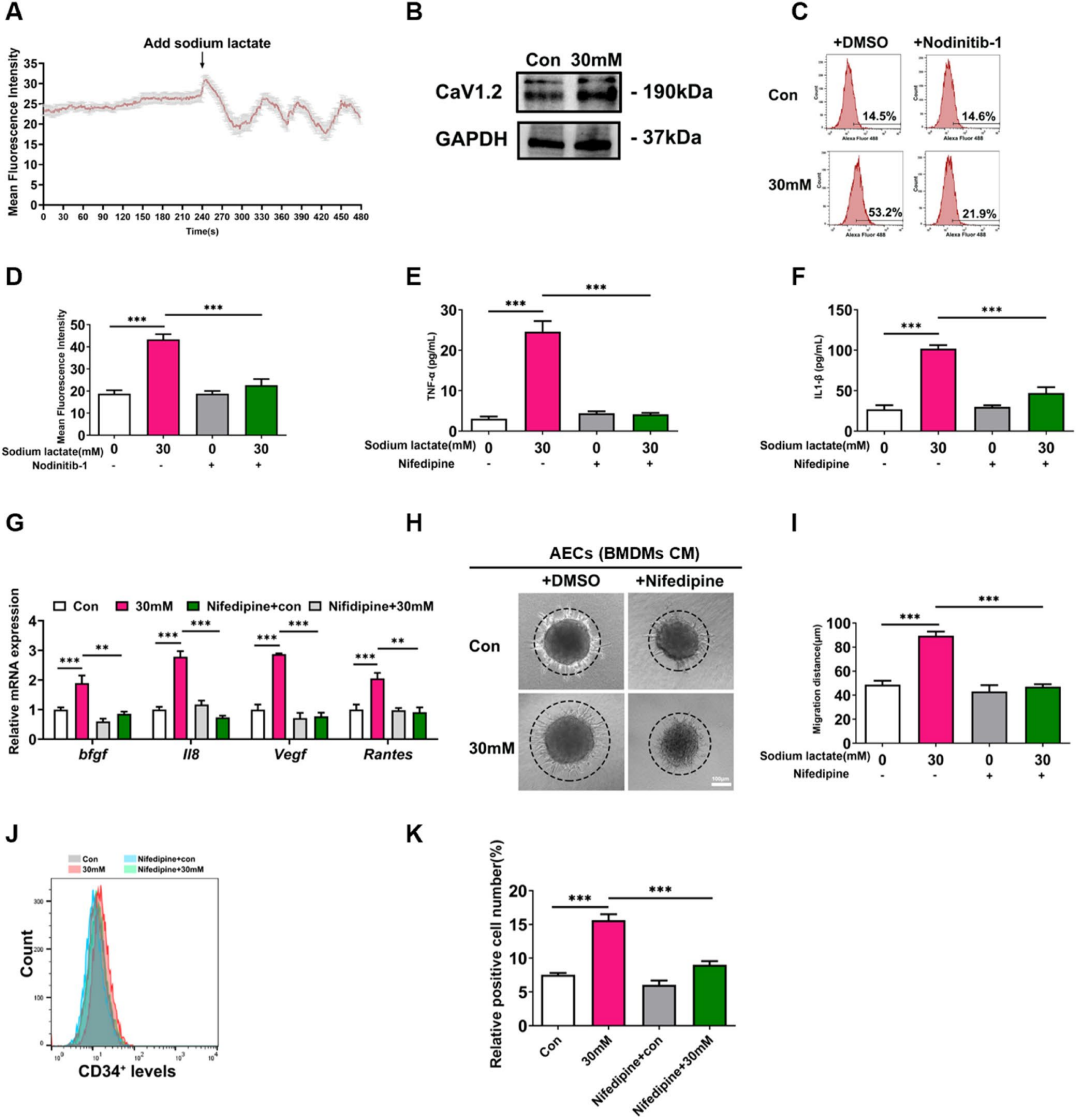
Lactate activates calcium influx in a NOD1‐dependent manner, which promotes M1 polarisation of BMDMs and enhances angiogenesis. (A) The line chart showed that the mean fluorescence intensity of Fluo‐4 labelled calcium ions varied with time. Calcium oscillation occurred when lactate was added at 240 s (*n* = 20). (B) Western blot analysis of CaV1.2 protein expression levels in BMDMs with or without lactate stimulation for 24 h. (C) Flow cytometry analysis of calcium ion concentration in BMDMs after pre‐incubation with or without Nodinitib‐1 for 2 h. (D) Quantification statistics of mean fluorescence intensity in (C) (*n* = 3). (E) ELISA assay of the TNFα protein expression level in BMDMs after pre‐treatment with or without nifedipine for 2 h (*n* = 3). (F) ELISA assay of the IL1β protein expression level in BMDMs after pre‐treatment with or without nifedipine for 2 h (*n* = 3). (G) RT‐qPCR of angiogenesis‐related gene expression levels after lactate treatment in the presence or absence of nifedipine (*n* = 3). (H) Spheroid sprouting experiment showed the migration distance of AECs treated with BMDMs CM with or without nifedipine. Scale bar: 100 μm. (I) Quantification statistics of the migration distance in (H) (*n* = 3). (J) Flow cytometry analysis of CD34 expression levels in AECs after treatment with BMDMs CM with or without nifedipine. (K) Quantification statistics of relative positive cell number in (J) (*n* = 3). All statistical data are presented as mean ± SEM. Samples were subjected to one‐way ANOVA. **p < 0.01, ***p < 0.001.

Elevated intracellular calcium ions are known to modulate macrophage M1 polarisation. The spatiotemporal variation of calcium ions in cytosol is manifested in the form of calcium oscillations or calcium waves [28]. On the basis of GO analysis, the BP term with increased calcium concentration was more enriched in the 30 mM group than the con group (Figure S3C). Furthermore, the Kyoto Encyclopedia of Genes and Genomes (KEGG) analysis identified calcium signalling pathway as being differentially expressed after 30 mM lactate treatment (Figure S4A). We subsequently used the Fluo‐4 AM fluorescence probe to track calcium mobilisation in BMDMs and the calcium oscillation was observed to be initiated immediately when lactate was added at 240 s, based on the line chart of the mean fluorescence intensity of calcium ions over time (Figure 4A, Movie S1).

Previous studies have shown that the NOD1 agonist elevates cytoplasmic levels of cyclic adenosine monophosphate (cAMP) [29], which promotes calcium influx by activating L‐type voltage‐gated calcium channels (VGCC) through protein kinase A (PKA) [30]. To confirm whether the calcium influx was the downstream effect that enables NOD1 response to lactate stimulation, we analyzed the PKA activity and protein levels of Cav1.2 based on expression profiles. The results showed that PKA activity was enhanced in lactate‐treated BMDMs and that the Cav1.2 subtype of the L‐type VGCC was significantly activated (Figures 4B and S4B). Next, we pretreated BMDMs with or without Nodinitib‐1 (NOD1 inhibitor), respectively, to verify whether lactate could stimulate the calcium influx by enhancing NOD1. The flow cytometry analysis demonstrated a significant increase in intracellular calcium concentration in BMDMs immediately after lactate administration, which was blocked by Nodinitib‐1 (Figure 4C,D). The results thus indicate that lactate activated calcium influx in a NOD1‐dependent manner, which ultimately triggers an increase in cytosolic calcium concentration.

Calcium ions are a ubiquitous intracellular signal responsible for controlling numerous cellular processes including activation of macrophage proinflammatory phenotype by regulating the secretion of inflammatory cytokines [31]. Therefore, BMDMs were pre‐treated with the L‐type calcium channel inhibitor nifedipine to block calcium influx, followed by lactate stimulation 2 h later, to validate the role of calcium influx in regulating M1 polarisation of BMDMs. Due to the presence of nifedipine, lactate was unable to increase the protein secretion of pro‐inflammatory factors (Figure 4E,F). Additionally, nifedipine inhibition of lactate‐promoted angiogenesis was also tested; the results showed that the administration of nifedipine attenuated the pro‐angiogenic effects of lactate with decreased levels of angiogenic marker genes (Figure 4G), shortened spheroid migration distance (Figure 4H,I), and downregulated expression of CD34 in AECs (Figure 4J,K). Collectively, the above data confirmed that lactate promoted M1 phenotype polarisation and the angiogenic process by activating NOD1‐dependent calcium influx.

### 3.5 Lactate Promotes Angiogenesis to Accelerate Bone Defect Repair

**FIGURE 5:**
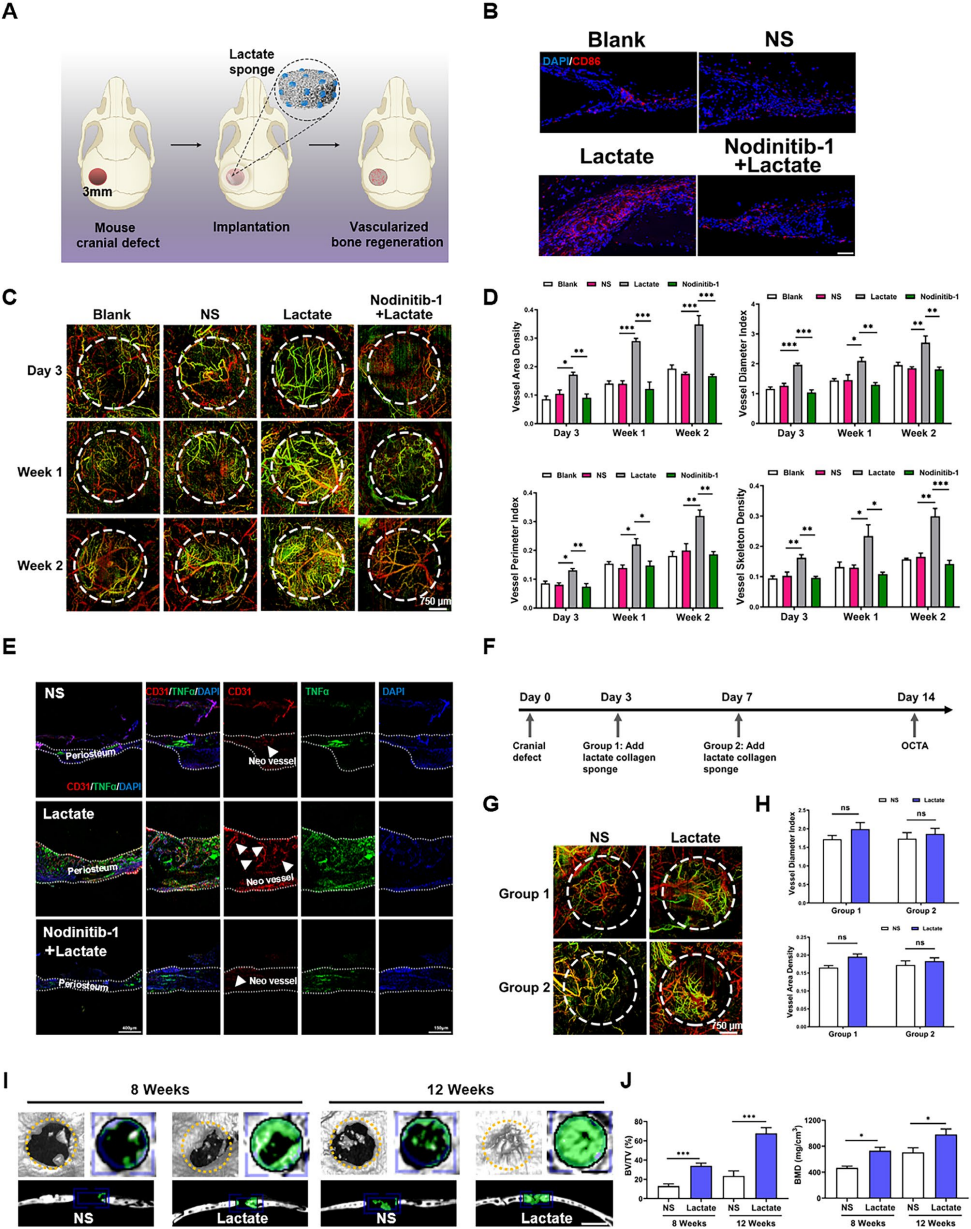
Lactate promotes angiogenesis to accelerate bone defect repair. (A) Schematic representation of collagen sponges impregnated with lactate being implanted into the cranial bone defect area. (B) Immunofluorescence staining images of CD86 on day 1 after bone defect surgery. Scale bar: 60 μm. (C) OCTA images depicting how the angiogenesis process changes with time in different treatment groups. Red colour indicates deep layer blood vessels (25–55 pixels), yellow colour indicates the middle layer (10–25 pixels), and green colour indicates the shallow layer (1–10 pixels). Scale bar: 750 μm. (D) Quantification analysis of vessel area density, vessel diameter index, vessel perimeter index, and vessel skeleton density in (C) (*n* = 3). (E) Co‐immunofluorescence staining of CD31 and TNFα expression in NS, Lactate and Nodinitib‐1 groups at 2 weeks after cranial defect surgery. Arrows indicate the newly‐formed blood vessels. Scale bar: Left 400 μm, right 150 μm. (F) Schematic illustration of delayed lactate addition at day 3 or day 7 after bone injury. (G) OCTA images depicting the effects of delayed addition of lactate on angiogenesis. Red colour indicates deep layer blood vessels (25–55 pixels), yellow colour indicates the middle layer (10–25 pixels), and green colour indicates the shallow layer (1–10 pixels). Scale bar: 750 μm. (H) Quantification analysis of the vessel area density and vessel diameter index in (G) (*n* = 3). Ns, no significance (*p* > 0.05). (I) Reconstructed micro‐CT images of mouse cranial bone at 8 and 12 weeks post‐surgery. The new bone was marked in green. Scale bar: Up 1500 μm, down 3000 μm. (J) Quantitative analysis of BV/TV and BMD values in the regenerated bone tissues (*n* = 6). All statistical data are presented as mean ± SEM. Samples were subjected to one‐way ANOVA and two‐tailed unpaired Student's *t*‐test. Ns, no significance (*p* > 0.05), *p < 0.05, **p < 0.01, ***p < 0.001.

To verify the efficacy of lactate in promoting vascularisation at bone defects and to explore optimal therapeutic strategies for targeting bone healing, a mouse cranial defect model was created. Collagen sponges were employed as a carrier of lactate to be delivered into the defects. The schematic diagram in Figure 5A showed the details of the implantation surgery (Figure 5A). Immunofluorescence staining showed that a greater accumulation of CD86‐labelled M1 macrophages was observed within the defect area in the lactate group 1 day post‐surgery, compared to the normal saline (NS) and blank groups. However, this effect was mitigated by Nodinitib‐1 treatment (Figure 5B), suggesting that lactate promoted macrophages polarisation towards the pro‐inflammatory M1 phenotype in bone defects.

Next, to evaluate the revascularisation process under the effect of lactate during the early stages of bone healing (day 3 to day 14 after creation of the bone defect), we performed in vivo imaging with optical coherence tomography angiography (OCTA) on day 3, day 7 and day 14 after surgery. The difference between mature and newly‐formed blood vessels is that the latter are thinner and looser in structure [32]. As a result, the lactate collagen sponge significantly accelerated revascularisation in bone defects from day 3 to day 14 with higher vessel area density, vessel skeleton density, vessel diameter index, and vessel perimeter index. In contrast, we discovered that implanting both Nodinitib‐1 and lactate collagen sponge counteracted the increase in revascularisation brought on by lactate alone (Figures 5C,D and S5A). The lactate group exhibited a significant increase in TNFα expression co‐localised with CD31, accompanied by new blood vessel formation, as shown by co‐immunofluorescence staining at 2 weeks post‐surgery compared to the NS group. These effects were abrogated in the presence of Nodinitib‐1, which further confirmed the concurrent spatiotemporal relationship between inflammation and vascularisation, with lactate playing a pivotal role in facilitating both responses during the bone defect repair process (Figure 5E).

It has been reported that the presence of M1 phenotype macrophages during the early phase of injury is essential for defect repair because of their ability to secrete high levels of VEGF [22]. In view of this, we investigated whether delayed addition of lactate would continue to trigger new blood vessel growth during the bone repair process. The lactate sponge was utilised for implantation into the bone defect site on the 3rd and 7th days after surgery, and the OCTA technique was performed on the 14th day to assess the outcome of angiogenesis (Figure 5F). It was observed that delayed lactate administration had no significant effect on angiogenesis (Figure 5G,H). These findings demonstrated the importance of lactate emergence timing and indicate that timely initiation of lactate intervention is necessary to promote M1 macrophage polarisation at the onset of bone defect.

Given the pro‐angiogenic effects of lactate, its influence on bone regeneration was assessed using a 3‐mm‐diameter mouse cranial defect model, which was implanted with Bio‐Oss Collagen, with or without lactate. Micro‐CT reconstructed images revealed that Bio‐Oss Collagen co‐treated with lactate (the Lactate group) facilitated faster bone healing at both the 8th and 12th weeks post‐surgery, with regenerated bone continuously growing from the edges of the defect towards its center over time. Although the NS group, which was not co‐treated with lactate, displayed almost no obvious bone formation at 8th weeks post‐surgery, with new bone gradually appearing only by 12th weeks (Figure 5I). H&E staining further confirmed these findings, demonstrating enhanced bone regeneration in the Lactate group at both time points, with the defect area increasingly filled with newly formed bone. In contrast, bone repair in the NS group was notably slower compared to the Lactate group (Figure S5B). Moreover, analyses of bone volume per tissue volume (BV/TV) and bone mineral density (BMD) values showed higher levels in the Lactate group than in the NS group after 8 and 12 weeks post‐surgery (Figure 5J). These results indicate that lactate enhanced the osteogenic effects of commercial bone filler materials and promoted bone defect regeneration in vivo, highlighting its therapeutic potential for accelerating bone healing.

Hence, our results suggest that lactate induced macrophage polarisation towards the pro‐inflammatory M1 phenotype, and the presence of M1 phenotype macrophages during the early phase of bone defect repair was essential for smooth progression of the healing process. Moreover, lactate treatment should be administered as early as possible in order to optimise its positive effects in promoting revascularisation and bone regeneration during the initial stages of bone defect healing.

## 4 Discussion

**FIGURE 6:**
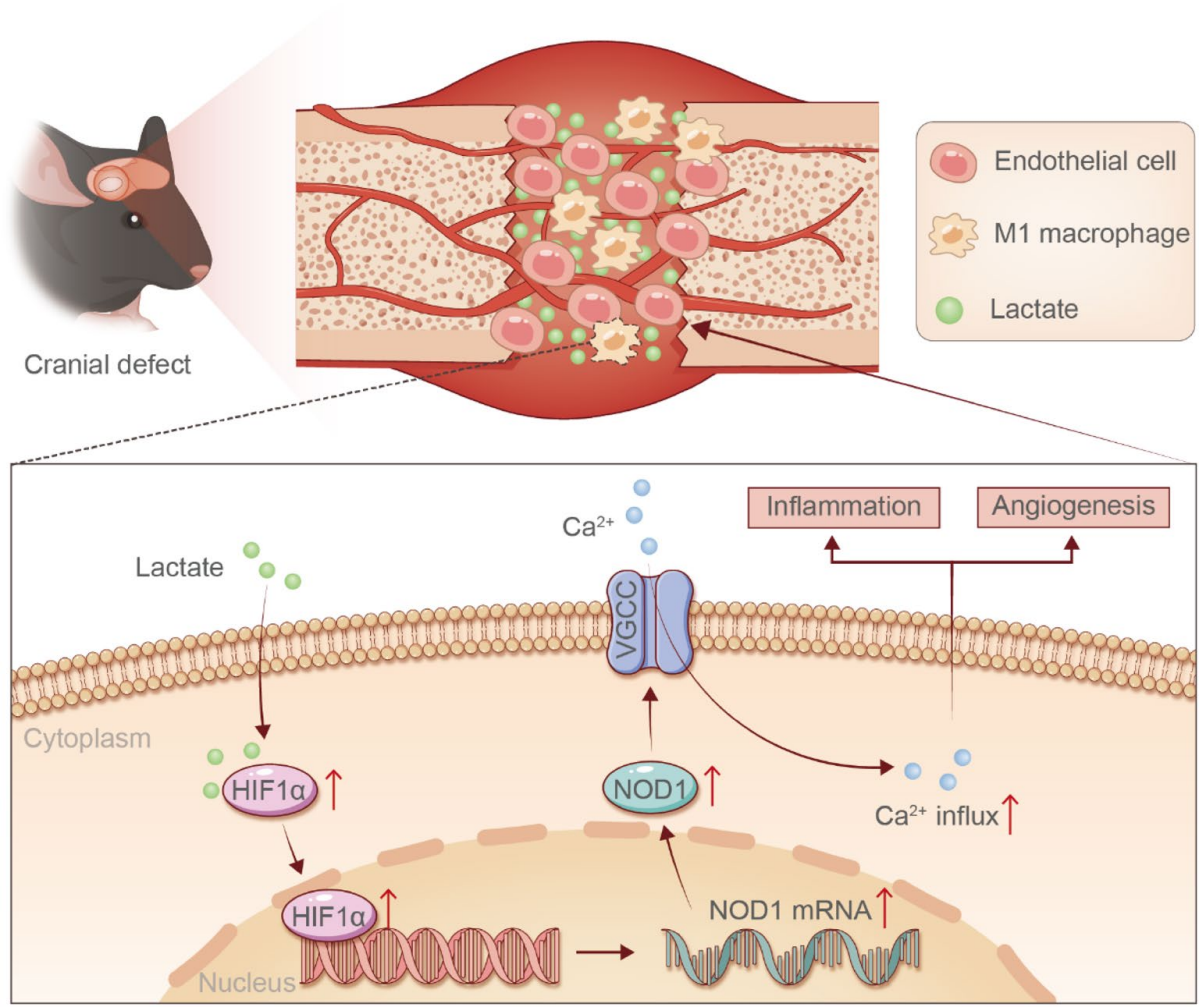
Schematic diagram illustrating the underlying mechanisms by which lactate promotes M1 polarisation of macrophages and early angiogenesis. During the early stage of bone defect healing, lactate accumulates and contributes to increasing NOD1 expression by stabilising HIF1α that in turn triggers a calcium influx, which ultimately polarises macrophages towards the M1 phenotype and accelerates vascularisation of endothelial cells.

M1 macrophages, typically induced by LPS or IFN‐γ during anti‐infectious responses, can also be driven by metabolic cues in the microenvironment. Our study revealed that elevated lactate levels in early bone defects stabilised HIF1α and upregulated NOD1, promoting M1 polarisation and subsequent tip cell sprouting and neovascularisation. In vivo, lactate administration further enhanced vascularised bone repair. Collectively, these findings identify lactate as a key metabolic regulator of macrophage activation and angiogenesis, offering a potential therapeutic strategy to accelerate vascularised bone regeneration (Figure 6).

Bone repair is orchestrated by a dynamic immune microenvironment involving coordinated actions of both innate and adaptive immune cells. In the early phase of injury, neutrophils and macrophages initiate inflammation, mediate debris clearance, and regulate cytokine signalling [33]. Subsequently, NK cells, macrophages, and dendritic cells promote angiogenesis and osteogenic differentiation through immunoregulatory cues [34]. During the adaptive immune phase, Th17 cells, γδ T cells, and regulatory T cells further shape the reparative niche by balancing inflammation and tissue regeneration [35, 36]. Immune metabolic signalling in the bone repair microenvironment plays a central role in regulating the fate of immune cells. Accumulating evidence indicates that lactate functions as an active immunometabolite, exerting broad regulatory effects on multiple immune cells. Recent studies have shown that lactate suppresses NK cell effector functions either indirectly or through direct inhibition of cytotoxic activity [37]. In neutrophils, lactate upregulates PD‐L1 expression via an MCT1‐dependent mechanism, thereby reducing apoptosis [38]. Moreover, lactate induces HIF‐1α‐dependent expression of NDUFA4L2 in dendritic cells, leading to suppression of autoreactive T cell responses [39]. Beyond these effects, lactate drives histone H3K18 lactylation in macrophages, upregulating genes such as *Arg1* and promoting a resolving phenotype in sepsis models [40]. Notably, lactate has recently been identified as a key metabolite enhancing γδ T17 responses and sustaining chronic inflammation through epithelial‐Th17 immune interactions [41]. Collectively, these findings underscore lactate as a key immunometabolic regulator shaping immune responses through metabolic and epigenetic mechanisms, providing a conceptual framework for understanding its role in the immune microenvironment of bone repair.

Over 99% of lactate dissociates into lactate anions and hydrogen ions, both biologically active [42]. In tumours, organic and inorganic acids promote M2‐like macrophage polarisation due to hypoxia and acidity [43, 44]. However, the role of lactate anions in macrophage regulation remains unclear. We hypothesise that lactate anions, rather than hydrogen ions, induce M1 polarisation. To test this, sodium lactate (5 mM and 30 mM) was applied to BMDMs, with 30 mM exhibiting marked effects. The 30 mM concentration has been widely recognised in studies investigating the influences of lactate on cellular function [45]. Furthermore, under inflammatory conditions such as arthritis, synovial fluid lactate concentrations have been reported to range from 10 to 40 mM [15]. Taken together, the above findings provide a physiological basis for in vitro experiments using 30 mM lactate to treat BMDMs and explore the mechanisms involved.

The NLR family, a key group of pattern recognition receptors (PRRs), senses diverse pathogen signals to activate immune defence [46]. Beyond their classical bacterial‐sensing functions, NOD1 and NOD2 also act as mediators of metabolic diseases, recognising metabolites in the microenvironment and modulating immune responses. Their unconventional metabolic roles involve direct signalling, stress response regulation, and inflammatory amplification [47]. However, their functions in the bone injury microenvironment remain unclear. Our study reveals that lactate specifically activates and upregulates NOD1, but not NOD2, in macrophages. Inhibition of NOD1 reverses lactate‐induced pro‐inflammatory and pro‐angiogenic effects, indicating that NOD1 is a critical regulator linking lactate metabolism to macrophage‐mediated inflammation and angiogenesis.

HIF1 is a transcriptional complex of α and β subunits, with HIF1β constitutively expressed and HIF1α continuously synthesised and degraded. Under normoxia, prolyl hydroxylase domain (PHD) hydroxylates HIF1α, leading to its ubiquitination and proteasomal degradation [48]. Lactate inhibits PHD activity, stabilising HIF1α, which is crucial for macrophage pro‐inflammatory activation and inflammation progression [49] and also promotes angiogenesis [50, 51]. Our results demonstrate that lactate stabilises HIF1α, enabling its binding to the NOD1 promoter and enhancing NOD1 transcription, thereby inducing macrophage pro‐inflammatory polarisation and angiogenic responses. Although HIF1α degradation can also be suppressed by PHD inactivation under hypoxia [26], we focus on the lactate‐driven HIF1α‐NOD1 signalling axis in tissue repair. Notably, lactate can independently stabilise HIF1α even under non‐hypoxic conditions, indicating its role as a metabolic signal initiating inflammation and angiogenesis in early bone injury.

The novelty of our study lies in demonstrating how the metabolite lactate drives M1 macrophage polarisation within the bone injury microenvironment via in vitro experiments. We further identify a novel role of NOD1 in sensing metabolic signals during bone repair. In vivo findings underscore the importance and timing of lactate intervention for vascularised bone regeneration. However, there are several limitations that need to be mentioned. First, this study primarily focused on macrophage polarisation, without considering the complex interplay among immune cell populations, which warrants future exploration. Second, we have revealed that lactate regulates macrophage polarisation and bone repair via the HIF1α‐NOD1‐calcium influx axis, wherein lactate activates Cav1.2 through the PKA pathway. As calcium signalling governs diverse cellular processes, including M1 polarisation [31], the precise downstream molecular mechanisms require further investigation in future studies.

## 5 Conclusion

Macrophage responses to bone injury signalling cues are critical to the bone healing process. However, the mechanisms of their polarisation towards the pro‐inflammatory phenotype have not yet been fully elucidated. Our in vitro experiments demonstrated that lactate induced macrophage polarisation towards the pro‐inflammatory M1 phenotype and enhanced angiogenic effects via the HIF1α‐NOD1‐calcium influx axis. Additionally, the in vivo experiments further validated that lactate administration promoted vascularised bone healing, whereas either the lack of lactate or delayed administration of lactate impaired revascularisation after bone injury. In summary, this study reveals that lactate promotes local macrophage polarisation to the pro‐inflammatory phenotype within bone defects and provides a new avenue for the possible application of lactate as a promising therapeutic target for enhancing vascularised bone regeneration.

## Author Contributions

**Lulu Liu:** data curation, formal analysis, investigation, writing – original draft. **Danning Ma:** data curation, formal analysis, methodology. **Jia Song:** data curation, formal analysis, writing – review and editing. **Boon Chin Heng:** writing – review and editing, funding acquisition. **Ying Huang:** data curation. **Xuehui Zhang:** data curation. **Mingming Xu:** conceptualisation, data curation. **Yan Wei:** data curation. **Tai Wei:** data curation, visualisation, funding acquisition, writing – review and editing. **Jinqi Wei:** conceptualisation, data curation, resources, supervision, funding acquisition. **Xuliang Deng:** conceptualisation, data curation, investigation, project administration, funding acquisition, supervision, validation.

## Funding

This work was supported by the National Natural Science Foundation of China 82221003, U22A20160 (to X.D.) and 82501103 (to T.W.), the National Key R&D Program of China 2022YFA1207304 (to X.D.), the Beijing Natural Science Foundation 7224350 (to T.W.), L242126 (to J.W.), the Beijing Natural Science Foundation, International Scientists Project IS23110 (to B.C.H.).

## Ethics Statement

The animal surgical procedure was approved by the Institutional Animal Care and Use Committee of Peking University (Approval number: LA2023047). All animal experiments were performed in the Laboratory Animal Science of Peking University Health Science Center.

## Conflicts of Interest

The authors declare no conflicts of interest.

## Supporting information


**Figure S1:** Histological staining images of cranial bone defects in mice. (A) The H&E staining images of cranial bone defects in mice at 24 h post‐injury. Scale bar: up 800 μm, down 200 μm. (B) Negative controls for IHC staining in cranial bone defect and intact bone. Scale bar: up 400 μm, down 100 μm.
**Figure S2:** Lactate induces M1 polarisation of macrophages and also promotes angiogenesis. The production of NO in BMDMs after 24 h of lactate treatment (*n* = 3). Results are presented as means ± SEM. Samples were subjected to one‐way ANOVA with Tukey's post hoc test. *p < 0.05, **p < 0.01.
**Figure S3:** Lactate promotes NOD1 expression by stabilising HIF1α to modulate M1 polarisation of BMDMs. (A) The heatmap of differentially expressed genes between the 30 mM and con groups. (B) The volcano plot of differentially expressed genes in the 30 mM group versus the con group. (C) GO enrichment analysis of differentially expressed genes between the 30 mM and con groups. (D) The mRNA expression of *NOD1* in the damage group and con group (*n* = 3). (E) Knockdown of *NOD1* by siRNA (*n* = 3). (F) Flow cytometry analysis of CD34 levels in AECs after treatment with BMDMs CM with or without Nodinitib‐1. All statistical data are presented as mean ± SEM. Samples were subjected to one‐way ANOVA and two‐tailed unpaired Student's *t*‐test. *p < 0.05, ***p < 0.001.
**Figure S4:** Lactate activates calcium influx in a NOD1‐dependent manner, which promotes M1 polarisation of BMDMs and enhances angiogenesis. (A) KEGG analysis of differentially expressed genes in BMDMs treated with or without 30 mM lactate for 24 h. (B) PKA activity in BMDMs with or without lactate treatment for 24 h (*n* = 3). All statistical data are presented as mean ± SEM. Samples were subjected to two‐tailed unpaired Student's *t*‐test. **p < 0.01.
**Figure S5:** Lactate promotes angiogenesis to accelerate bone defect repair. (A) OCTA images depicting how the angiogenesis process changes with time in different treatment groups. Red colour indicates deep layer blood vessels (25–55 pixels), yellow colour indicates the middle layer (10–25 pixels), and green colour indicates the shallow layer (1–10 pixels). Scale bar: 375 μm. (B) H&E staining images of the cranial defects after 8 weeks and 12 weeks post‐implantation respectively. Scale bar: 500 μm.
**Table S1:** Primer sequences for RT‐qPCR analysis.
**Table S2:** siRNA sequences for transfection.

**Movie S1:** Effects of lactate on calcium mobilisation labelled by Fluo‐4 AM probe in BMDMs (*n* = 20, *t* = 240 s).

## Data Availability

The data that support the findings of this study are available from the corresponding author upon reasonable request.
