# Supplementary material for: Lactate Accelerates Early Angiogenesis and Bone Regeneration Through Macrophage M1 Polarisation
Source: Cell Prolif. 2026 Jan 26;59(7):e70177. doi: 10.1111/cpr.70177 (PMC13325498; doi:10.1111/cpr.70177)
Supplement: Supplementary file 1 — Figure S1: Histological staining images of cranial bone defects in mice. (A) The H&E staining images of cranial bone defects in mice at 24 h post‐injury. Scale bar: up 800 μm, down 200 μm. (B) Negative controls for IHC staining in cranial bone defect and intact bone. Scale bar: up 400 μm, down 100 μm. Figure S2: Lactate induces M1 polarisation of macrophages and also promotes angiogenesis. The production of NO in BMDMs after 24 h of lactate treatment (n = 3). Results are presented as means ± SEM. Samples were subjected to one‐way ANOVA with Tukey's post hoc test. *p < 0.05, **p < 0.01. Figure S3: Lactate promotes NOD1 expression by stabilising HIF1α to modulate M1 polarisation of BMDMs. (A) The heatmap of differentially expressed genes between the 30 mM and con groups. (B) The volcano plot of differentially expressed genes in the 30 mM group versus the con group. (C) GO enrichment analysis of differentially expressed genes between the 30 mM and con groups. (D) The mRNA expression of NOD1 in the damage group and con group (n = 3). (E) Knockdown of NOD1 by siRNA (n = 3). (F) Flow cytometry analysis of CD34 levels in AECs after treatment with BMDMs CM with or without Nodinitib‐1. All statistical data are presented as mean ± SEM. Samples were subjected to one‐way ANOVA and two‐tailed unpaired Student's t‐test. *p < 0.05, ***p < 0.001. Figure S4: Lactate activates calcium influx in a NOD1‐dependent manner, which promotes M1 polarisation of BMDMs and enhances angiogenesis. (A) KEGG analysis of differentially expressed genes in BMDMs treated with or without 30 mM lactate for 24 h. (B) PKA activity in BMDMs with or without lactate treatment for 24 h (n = 3). All statistical data are presented as mean ± SEM. Samples were subjected to two‐tailed unpaired Student's t‐test. **p < 0.01. Figure S5: Lactate promotes angiogenesis to accelerate bone defect repair. (A) OCTA images depicting how the angiogenesis process changes with time in different treatment groups. Red colour indic [file CPR-59-e70177-s002.docx]

Supplementary Materials

**Lactate Accelerates Early Angiogenesis and Bone Regeneration through Macrophage M1 Polarization**

Authors: *Lulu Liu ^1^, Danning Ma ^1^, Jia Song ^2^, Boon Chin Heng ^2^, Ying Huang ^3^, Xuehui Zhang ^2^, Mingming Xu ^3^, Yan Wei ^3^, Tai Wei ^1*^, Jinqi Wei ^1*^, Xuliang Deng ^3*^*

Affiliations:

Lulu Liu, Danning Ma, Tai Wei, Jinqi Wei

1 First Clinical Division, Peking University School and Hospital of Stomatology & National Center of Stomatology & National Clinical Research Center for Oral Diseases & National Engineering Research Center of Oral Biomaterials and Digital Stomatology & Research Center of Engineering and Technology for Computerized Dentistry Ministry of Health & NMPA Key Laboratory for Dental Materials; No.37A, Xishiku Avenue, Xicheng District, Beijing, 100034, PR China.

Jia Song, Boon Chin Heng, Xuehui Zhang

2Department of Dental Materials & Dental Medical Devices Testing Center, Peking University School and Hospital of Stomatology & National Center of Stomatology & National Clinical Research Center for Oral Diseases & National Engineering Research Center of Oral Biomaterials and Digital Stomatology & Research Center of Engineering and Technology for Computerized Dentistry Ministry of Health & NMPA Key Laboratory for Dental Materials; No.22,Zhongguancun South Avenue, Haidian District, Beijing, 100081, PR China.

Ying Huang, Mingming Xu, Yan Wei, Xuliang Deng

3Department of Geriatric Dentistry, Peking University School and Hospital of Stomatology & National Center of Stomatology & National Clinical Research Center for Oral Diseases & National Engineering Research Center of Oral Biomaterials and Digital Stomatology & Research Center of Engineering and Technology for Computerized Dentistry Ministry of Health & NMPA Key Laboratory for Dental Materials; No.22, Zhongguancun South Avenue, Haidian District, Beijing, 100081, PR China.

Corresponding: weitai@bjmu.edu.cn (T. W.); weijinqipkuss@bjmu.edu.cn (J. W.); kqdengxuliang@bjmu.edu.cn (X. D.).

Keywords: lactate; bone regeneration; angiogenesis; M1 polarization; macrophages

**
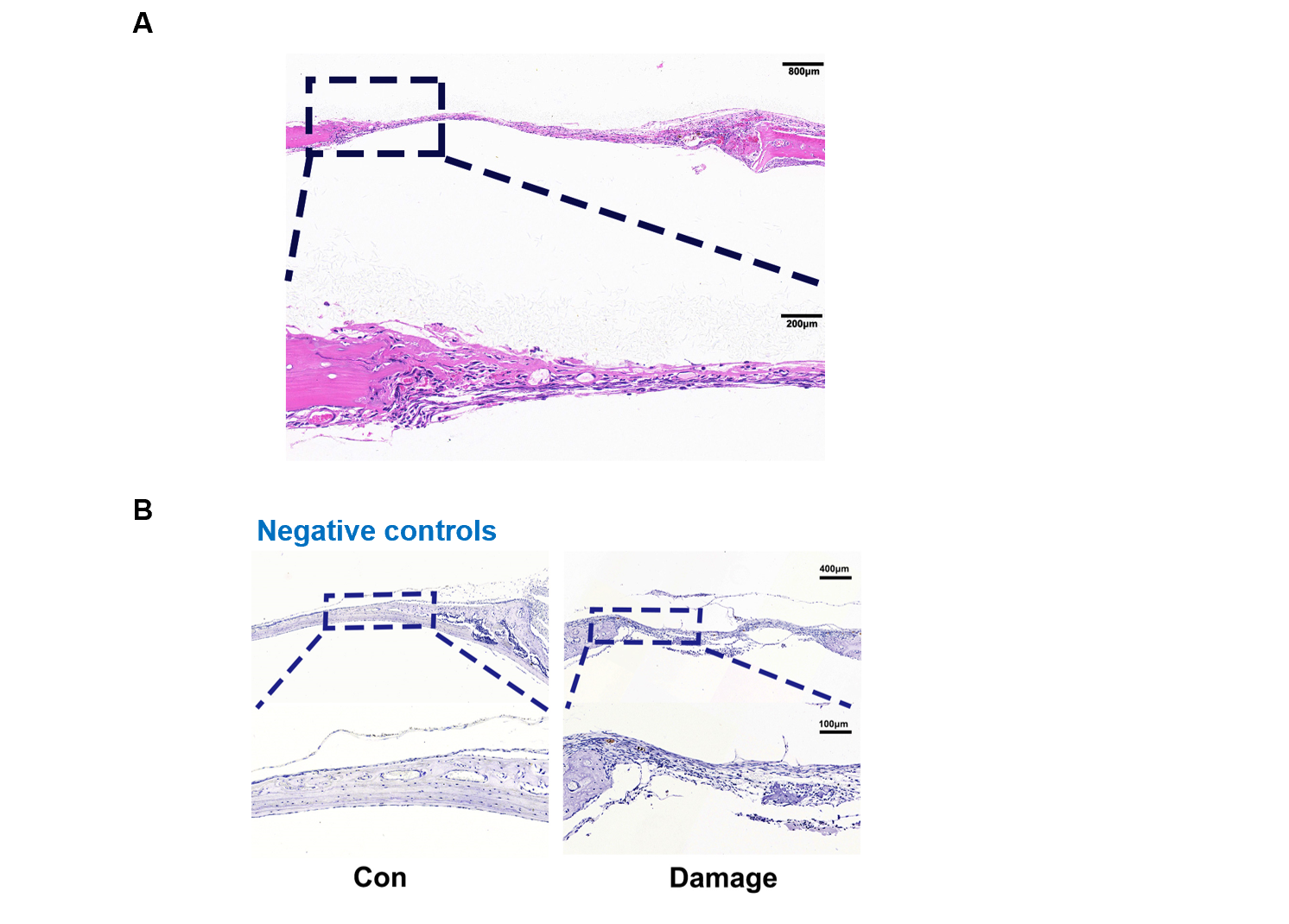
**

**Figure S1. Histological staining images of cranial bone defects in mice.**

**(A)** The H&E staining images of cranial bone defects in mice at 24 h post-injury. Scale bar: up 800 μm, down 200 μm.

**(B)** Negative controls for IHC staining in cranial bone defect and intact bone. Scale bar: up 400 μm, down 100 μm.


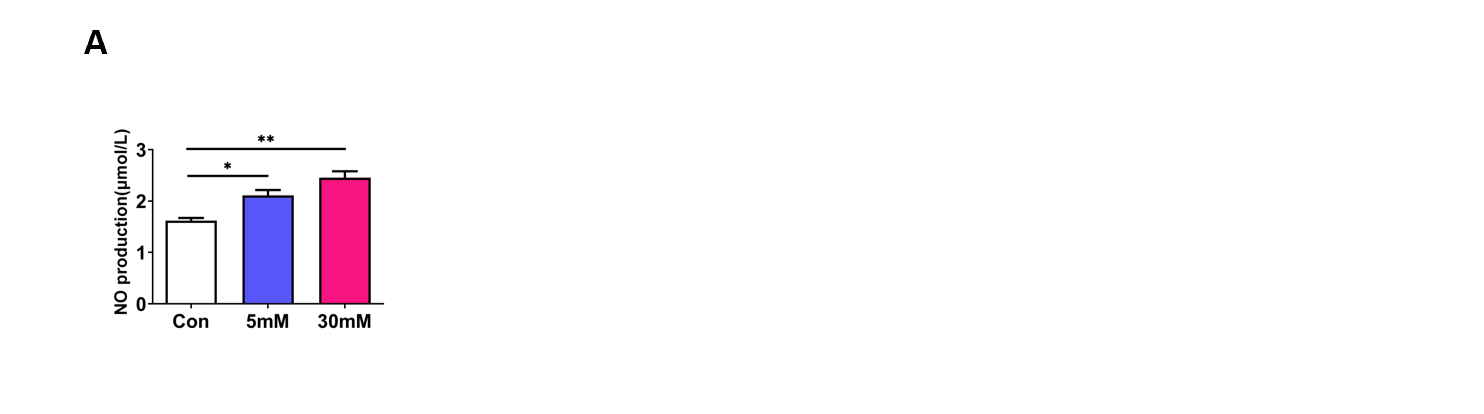


**Figure S2. Lactate induces M1 polarization of macrophages and also promotes angiogenesis.**

The production of NO in BMDMs after 24 h of lactate treatment (*n* = 3).

Results are presented as means ± SEM. Samples were subjected to one-way ANOVA with Tukey’s post hoc test. **p* < 0.05, ***p* < 0.01.


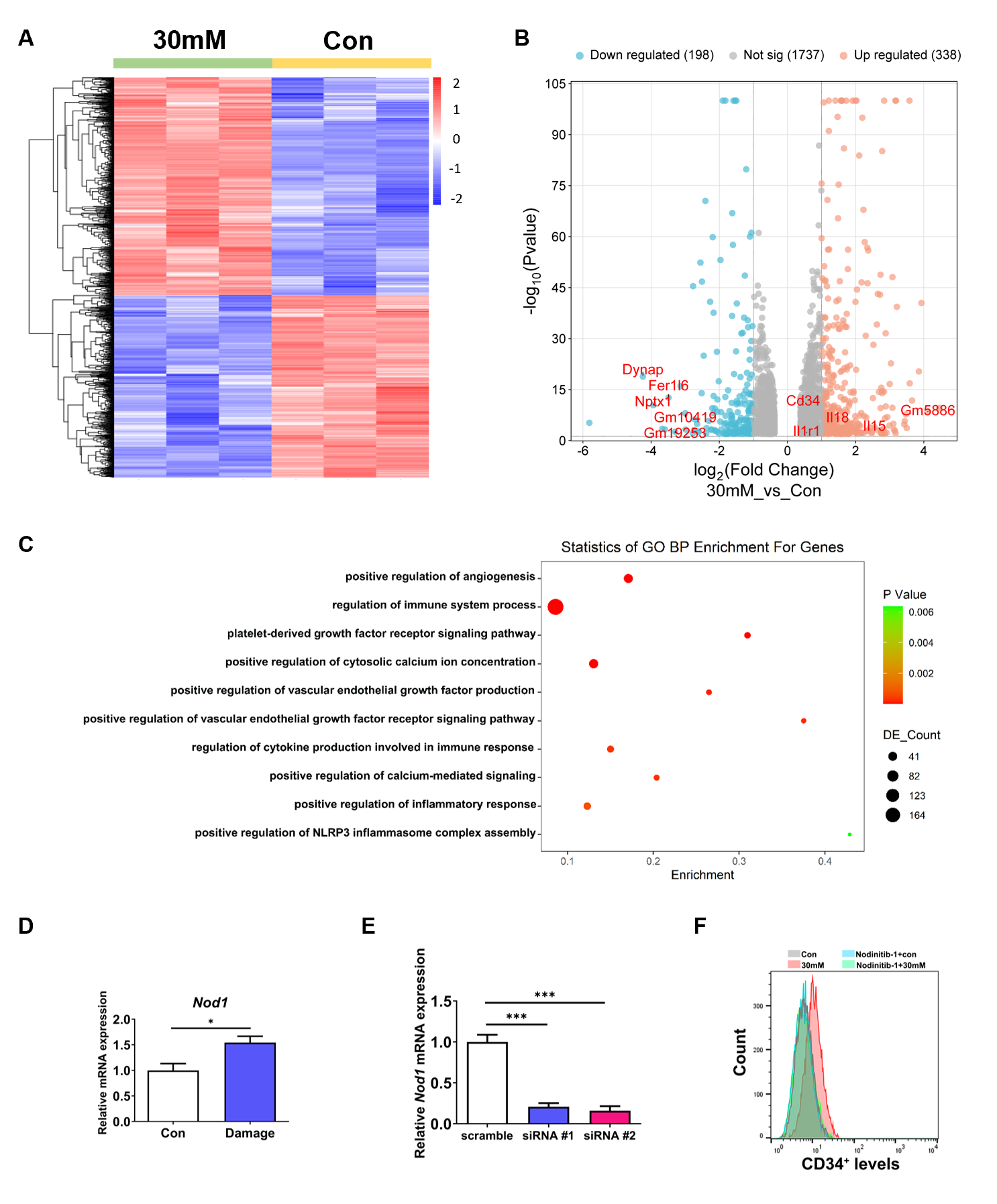


**Figure S3. Lactate promotes NOD1 expression by stabilizing HIF1α to modulate M1 polarization of BMDMs.**

**(A)** The heatmap of differentially expressed genes between the 30 mM and con groups.

**(B)** The volcano plot of differentially expressed genes in the 30 mM group versus the con group.

**(C)** GO enrichment analysis of differentially expressed genes between the 30 mM and con groups.

**(D)** The mRNA expression of *Nod1* in the damage group and con group (*n* = 3).

**(E)** Knockdown of *Nod1* by siRNA (*n* = 3).

**(F)** Flow cytometry analysis of CD34 levels in AECs after treatment with BMDMs CM with or without Nodinitib-1.

All statistical data are presented as mean ± SEM. Samples were subjected to one-way ANOVA and two-tailed unpaired Student’s t-test. **p* < 0.05, ****p* < 0.001.

**
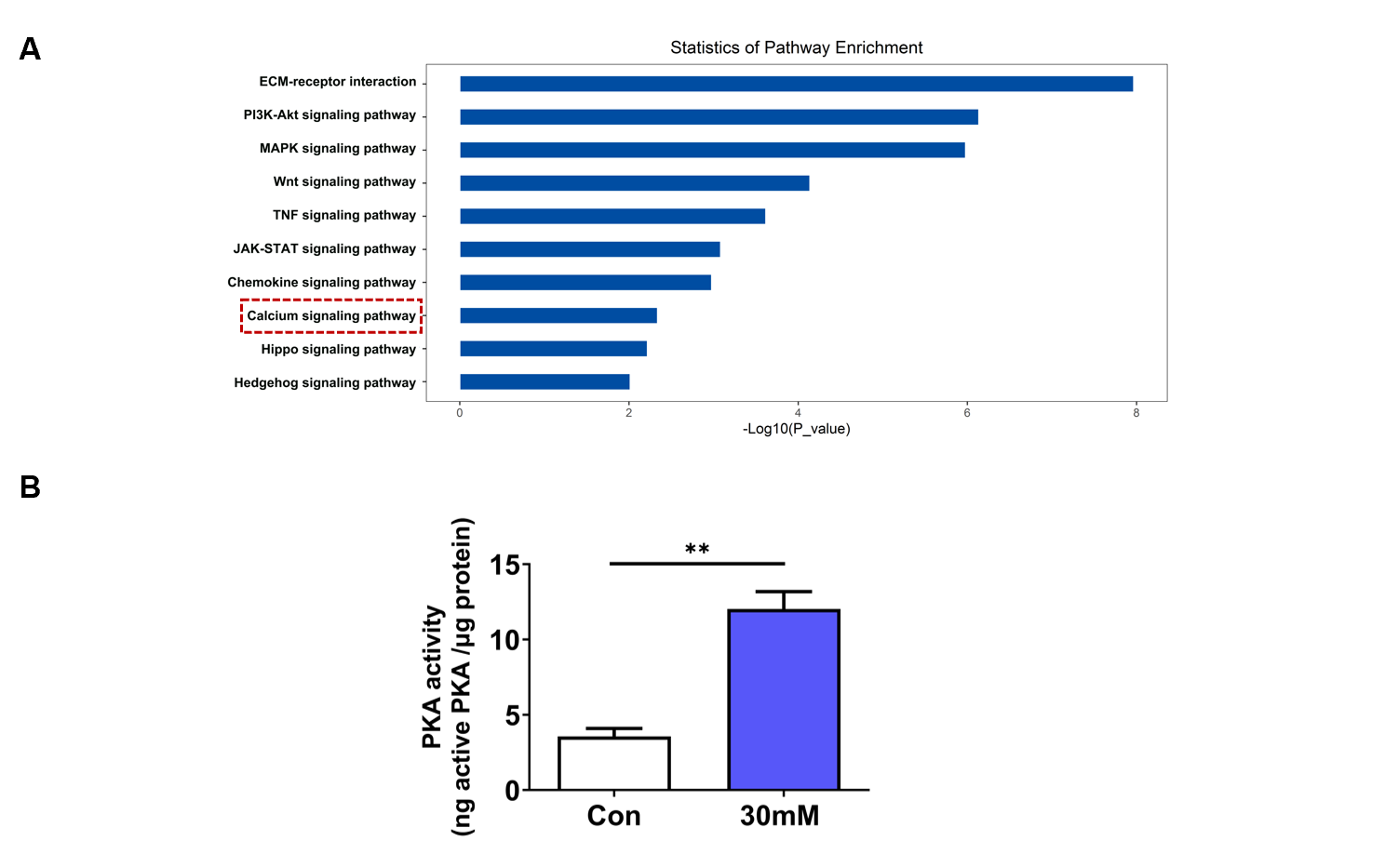
**

**Figure S4.** **Lactate activates calcium influx in a NOD1-dependent manner, which promotes M1 polarization of BMDMs and enhances angiogenesis.**

**(A)** KEGG analysis of differentially expressed genes in BMDMs treated with or without 30 mM lactate for 24 h.

**(B)** PKA activity in BMDMs with or without lactate treatment for 24 hours (*n* = 3).

All statistical data are presented as mean ± SEM. Samples were subjected to two-tailed unpaired Student’s t-test. ***p* < 0.01.


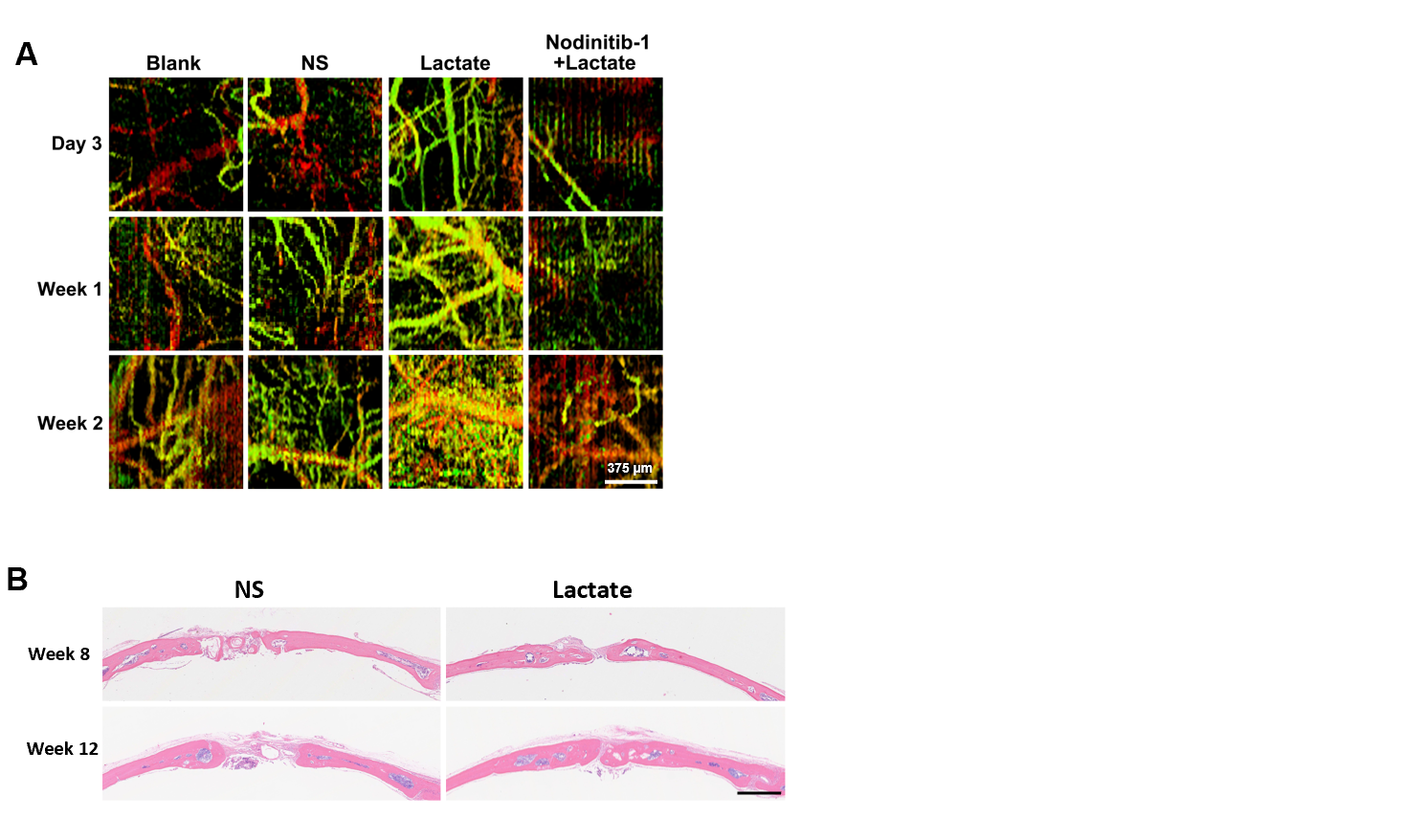


**Figure S5. Lactate promotes angiogenesis to accelerate bone defect repair.**

**(A)** OCTA images depicting how the angiogenesis process changes with time in different treatment groups. Red color indicates deep layer blood vessels (25-55 pixels), yellow color indicates the middle layer (10-25 pixels), and green color indicates the shallow layer (1-10 pixels)**.** Scale bar: 375 μm.

**(B)** H&E staining images of the cranial defects after 8 weeks and 12 weeks post-implantation respectively. Scale bar: 500 μm.

**Table S1 | Primer sequences for RT-qPCR analysis**

| Target gene | Forward sequence (5’ - 3’) | Reverse sequence (5’ - 3’) |
| --- | --- | --- |
| *Ccr7* | AGGCTCAAGACCATGACG | CACAGGTAGACGCCAAAGA |
| *inos* | GCCCAGGAGGAGAGAGAT | GCAAAGAGGACTGTGGCT |
| *Tnfα* | CGCTGAGGTCAATCTGC | GGCTGGGTAGAGAATGGA |
| *Il1β* | TGAGGACATGAGCACCTTC | GGGAACGTCACACACCA |
| *Cd163* | TGCTGTCACTAACGCTCCTG | TCATTCATGCTCCAGCCGTT |
| *Il10* | GCCCTTTGCTATGGTGTC | TCTCCCTGGTTTCTCTTCC |
| *Il8* | ATCTTCGTCCGTCCCTGTGA | TTCACCCATGGAGCATCAGG |
| *Vegf* | GGAAACCAGCAGAAAGAGG | CCCAAAAGCAGGTCACTC |
| *Rantes* | TGCTGCTTTGCCTACCTCTC | TCTTCTCTGGGTTGGCACAC |
| *bfgf* | CCATTGTCCCAGTAAAGAAAA | AATAAAGCAAATGCGTGAAAA |
| *Nod1* | GACAAGTGCCCTCCATCCTTTA | TTTGACCACATGTGTCTCGCT |
| *Nod2* | TGTCCAACAATGGCATCACCT | TTCCCTCGAAGCCAAACCTC |
| *Gapdh* | AAGAAGGTGGTGAAGCAGG | GAAGGTGGAAGAGTGGGAGT |

**Table S2 | siRNA sequences for transfection**

| siRNA-#1 | CCAAAGTCTTCGAGAATGA |
| --- | --- |
| siRNA-#2 | GGGTGAAGGTGCTATGTGA |

**Supplementary movie**

Effects of lactate on calcium mobilization labeled by Fluo-4AM probe in BMDMs (*n* = 20, *t* = 240 s).
